# Structure and Dynamics of Imidazolium in an Ionic Liquid‐PEGDA Iongel via IR, 2D‐IR, and NMR Spectroscopy

**DOI:** 10.1002/cphc.202500925

**Published:** 2026-04-19

**Authors:** Kallol Mukherjee, Matthew R. Liberatore, Tyler A. Parrack, Sean Garrett‐Roe

**Affiliations:** ^1^ Department of Chemistry University of Pittsburgh Pittsburgh Pennsylvania USA

**Keywords:** 2D‐IR spectroscopy, C‐D stretching mode, imidazolium, iongel, PEGDA

## Abstract

Fourier transform infrared absorption spectroscopy, ultrafast two‐dimensional infrared spectroscopy, nuclear magnetic resonance spectroscopy, and density functional theory calculations show that, in iongels composed of 1‐ethyl‐3‐methylimidazolium bis(trifluoromethylsulfonyl)imide ([C2C1Im][Tf2N]) and cross‐linked poly(ethylene glycol)diacrylate (cl‐PEGDA), imidazolium cations solvate PEGDA chains in tight and loose complexes. H/D isotope substitution at the 2‐position of the imidazolium ring simplifies the CH‐stretching vibrational bands. Tight complexes are characterized by stronger, directional hydrogen bonding interactions between the 2‐position of the imidazolium ring and the ethereal oxygens. The loose complexes resemble bulk ionic liquid nonspecifically perturbed by the PEGDA chains.

## Graphical abstract

Linear and nonlinear vibrational spectroscopies detect tight and loose complexes between imidazolium cations and poly(ethyleneoxide) chains in an iongel.

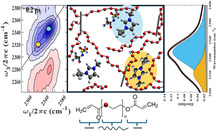

## 1 Introduction

Civilization faces a grand challenge—to reduce or reverse global warming [1, 2]. Burning fossil fuels, industrial processes, and some agricultural activities increase the amount of greenhouse gases, like carbon dioxide (${\mathrm{CO}}_{2}$), methane (${\mathrm{CH}}_{4}$), and nitrous oxide ($N_{2}O$), in the atmosphere and ultimately drive global warming [3]. Renewable energy sources and biofuels are meaningful ways to mitigate the generation of greenhouse gases. Nevertheless, post‐combustion carbon capture and storage could help decarbonize industries reliant on fossil fuels and, eventually, capture ${\mathrm{CO}}_{2}$ directly from the atmosphere. Membrane‐based separation of ${\mathrm{CO}}_{2}$ could provide lower cost, low footprint, and energy efficiency [4]. Ionic liquid (IL) and polymer composite membranes (iongels [5]) are emerging as alternatives to polymer membranes. Some of these membranes successfully surpass the Robeson upper bound [6, 7, 8, 9]. The Robeson upper bound is an empirical relation between the gas permeability and the gas selectivity of a gas separation membrane‐material, and it helps to understand the trade‐off between these two properties for each separation membrane [6, 7]. A clear, molecular‐level understanding of the structure and dynamics of iongels could reveal the origin of the measured permselectivity (both the thermodynamic factor of solubility and the dynamic factor of permeability).

**SCHEME 1:**
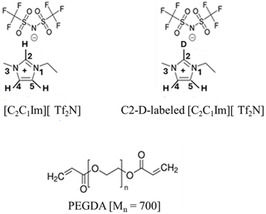
Chemical structures of [C2C1Im][Tf2N] (H/D) and PEGDA.

The iongel 1‐ethyl‐3‐methylimidazolium bis(trifluoromethylsulfonyl)imide ([C2C1Im][Tf2N], Scheme 1) in cross‐linked poly(ethylene glycol) diacrylate (cl‐PEGDA, Scheme 1) is a promising system to build the basic understanding about this class of materials. Imidazolium is the archetypal cation for modern ILs [10]. Dialkylimidazolium salts, in particular, have low melting temperatures, $T_{m}$, due to the asymmetry of the alkyl sidechains, which are typically a methyl and an ethyl group. The charge on the imidazolium ring polarizes the C–H bond at the 2‐position sufficiently for it to become a slightly acidic and a weak hydrogen bond donor; the 4‐ and 5‐positions are also polarized, but less so. The ${\left[{\mathrm{Tf}}_{2}N\right]}^{-}$ anion has a high affinity for ${\mathrm{CO}}_{2}$ and a low viscosity (∼50 mPa s, at least compared to other ILs, e.g., ${\left[{\mathrm{PF}}_{6}\right]}^{-}$ (200 mPa s), which together make it promising for ${\mathrm{CO}}_{2}$ capture applications [11]. PEGDA is a commercially available polymer that can be easily cross‐linked (thermally or photocatalytically) in the presence of an IL to form iongels. When cross‐linked, the acrylate groups form rigid polyacrylate rods interconnected by flexible poly(ethylene glycol) (also known as poly(ethylene oxide) (PEO)) chains [12]. Polyethers, in general, have been noted for their high solubility of ${\mathrm{CO}}_{2}$—being ‘${\mathrm{CO}}_{2}$‐philic’—which is hypothesized to be the result of attraction between the Lewis acidic carbon of ${\mathrm{CO}}_{2}$ and the Lewis basic ether oxygens [13, 14]. Pure PEO is semicrystalline, and the acrylate cross‐linkers [13] and IL [15] both disrupt PEO crystallization and improve membrane performance.

The wide miscibility ranges and high thermal stabilities [15] allow us to explore a broad range of IL‐PEGDA compositions. [C2C1Im][Tf2N] is less hygroscopic than most other imidazolium‐based ILs, which reduces the uncontrolled water content. The IL enhances the ${\mathrm{CO}}_{2}$ permeability through the PEGDA matrix by plasticizing the polymer network. As IL is added to PEGDA, the permeability increases from 100 to 500 barrer while the ${\mathrm{CO}}_{2}$/$N_{2}$ selectivity drops from 60 to 30, leading to an overall improvement in permselectivity of a factor of 2.5 [16]. The permeability of ${\mathrm{CO}}_{2}$ in these iongels increases with shorter alkyl side‐chains on the imidazolium cations and decreases with the cross‐linking density [16, 17]. Not all imidazolium‐based ILs, however, exhibit good miscibility with the PEGDA matrix. Imidazolium‐based ILs with soft anions and acidic protons appear more compatible than the others [15]. Recent reports show that an imidazolium‐based iongel can even cross the Robeson upper‐limit for ${\mathrm{CO}}_{2}$/$N_{2}$ gas mixtures [5, 18], although why this iongel performs so well is not clear yet.

The interaction of the IL with the PEGDA matrix is critical for the physical and chemical performance of the iongels. Simulation studies on PEO chains in imidazolium‐based ILs [19, 20, 21] suggest that the imidazolium cation interacts specifically with the ether oxygens, and two different solvation motifs are predicted. In the first, the PEO chains are extended and diffusely solvated by the IL. In the second, the PEO chains collapse and form ring‐like segments that wrap around the imidazolium [19, 20, 21]. These atomistic models are supported by some small angle neutron scattering measurements [22] but not others [23]. Analogous hydrogen bonded complexes between imidazolium and certain crown ethers can be crystallized [24]. No direct experimental evidence to support or refute the specific interaction between imidazolium and the PEO chains, however, is available.

Vibrational spectroscopy is a powerful technique to understand intermolecular interactions [25]. Raman, mid‐IR, and far‐IR vibrational assignments in ILs and solutions have been recently reviewed [26]. Two‐dimensional infrared (2D‐IR) spectroscopy measures the structural and dynamical changes on an ultrafast time scale [27] and has been applied to ILs [28, 29, 30], IL mixtures [31, 32, 33, 34], ${\mathrm{CO}}_{2}$ absorption [35, 36, 37, 38, 39, 40], SILMs [41], thin‐films [32, 42], and interfaces [43, 44, 45]. A recent study on vibrational dynamics of ${\mathrm{CO}}_{2}$ in an iongel system reveals that ${\mathrm{CO}}_{2}$ experiences a significantly different environment in the 50% by volume (% v/v) mixture than the parent compounds, and it occupies the interfacial domains in the mixture [46]. A fundamental understanding of the interaction and dynamics of ILs in these composite materials will provide chemical principles for the design and optimization of these carbon capture materials.

In this study, we have used the CH‐stretching modes of the imidazolium ring at the 2‐, 4‐, and 5‐positions, $C_{\left(2\right)}H$ and $C_{\left(4,5\right)}H$, as vibrational probes to investigate the [C2C1Im][Tf2N]/cl–PEGDA interaction at different [C2C1Im][Tf2N] loadings. Both the IL and the PEGDA have several IR‐active modes [18, 47]. The aliphatic CH‐stretching modes, 2700 ${\mathrm{cm}}^{-1}$ to 3050 ${\mathrm{cm}}^{-1}$, have low oscillator strengths and are insensitive to their local environment. The carbonyl stretches of the PEGDA also are insensitive to interactions with the IL. The aromatic CH‐stretches, however, prove to be useful probes of the interaction of imidazolium and PEGDA.

The CH‐stretching modes of imidazolium are very sensitive to the hydrogen bonding interactions between IL cation and the anion, especially at the 2‐position [48, 49, 50]. Generally, hydrogen bonding increases the anharmonicity of the CH‐stretch and redshifts the vibrational band [51]. For example, as the size of a halide anion decreases, the hydrogen bond strength increases, and the CH‐stretch shifts to lower wavenumbers [49]. Similarly, hydrogen bonding with co‐solvents can shift the CH‐stretching frequency of the cation to either higher [52, 53, 54, 55] or lower wavenumbers [56], depending on the basicity of the co‐solvent.

The proper assignment of the aromatic CH‐stretching bands (3100 to 3200 ${\mathrm{cm}}^{-1}$) of [C2C1Im][Tf2N] has been debated. Multiple peaks appear in the IR spectrum due to the ring CH‐stretches. One proposal is that the high frequency features (> 3140 ${\mathrm{cm}}^{-1}$) are the $C_{\left(4\right)}H$ and $C_{\left(5\right)}H$ symmetric and antisymmetric stretch absorption bands and the low frequency features are bands due to the $C_{\left(2\right)}H$ stretch [47, 57, 58]. Another proposal is $C_{\left(2\right)}H$, $C_{\left(4\right)}H$, and $C_{\left(5\right)}H$ all contribute to the high frequency features (> 3140 ${\mathrm{cm}}^{-1}$) and that the low frequency features (3100 to 3140 ${\mathrm{cm}}^{-1}$) are Fermi resonances of overtones and combination bands of N‐C–H ring bending modes at ∼1570 ${\mathrm{cm}}^{-1}$) with the bright CH‐stretches [59, 60]. The agreement of cluster experiments and high‐level anharmonic vibrational frequency calculations strongly supports the conclusion that Fermi resonances are the origin of the complex spectral structure [60]. Regardless, this complex band shape impedes direct interpretation of the CH‐stretching region.

H/D isotope substitution at the 2‐position simplifies the vibrational spectrum [59, 60]. Because the proton at the 2‐position is more acidic than at the 4‐ and 5‐positions, mild conditions are sufficient to selectively exchange the labile proton for a deuteron. The $C_{\left(2\right)}D$ stretching vibration absorbs in a single band spectrally isolated from other interfering modes (2,300 to 2,400 ${\mathrm{cm}}^{-1}$). Isotopic substitution also provides an opportunity to simultaneously observe the $C_{\left(2\right)}D$ stretching region, and the $C_{\left(4,5\right)}H$ stretching regime for imidazolium ring without changing the chemical structure of the cation, in contrast to methylation of the 2‐position. The $C_{\left(2\right)}D$ stretching mode can report interactions and dynamics in ILs and IL‐organic solvent mixtures [61, 62, 63]. This mode captures the specific interactions between the IL and organic solvent reasonably well [61]. Though its molar extinction coefficient is low for 2D‐IR spectroscopy (∼70 $M^{-1}\;{\mathrm{cm}}^{-1}$ [51, 64, 65]), it is high compared to an aliphatic CH‐stretch (∼1 $M^{-1}\;{\mathrm{cm}}^{-1}$), and it can be prepared at high enough concentration to be a useful vibrational chromophore. Though its vibrational lifetime is short (< 1ps) [61], it can report the degree of structure inhomogeneity and local structural fluctuations [63].

NMR spectroscopy also captures the intermolecular interactions and dynamics in ILs and has been reviewed recently [66]. H‐bonding interactions [47, 49, 67, 68] and ion dynamics [69, 70, 71] of pure ILs and their mixtures have been studied extensively using multinuclear one and two‐dimensional NMR spectroscopy.

In this work, we show how the imidazolium cations interact with PEGDA, how it is different at the 2‐ and 4‐/5‐positions, and how it changes as a function of the iongel composition and cross‐linking. Linear FTIR spectroscopy probes the C–H/D ring modes as a function of PEGDA volume fraction. 2D‐IR spectroscopy of the $C_{\left(2\right)}D$ band shows the ultrafast dynamics of the cation. Composition‐dependent NMR and IR measurements show how differently the ring hydrogens interact with the PEGDA chains. Density functional theory supports the assignments and interpretation.

## 2 Results and Discussion

### 2.1 FTIR Spectroscopy

**FIGURE 1:**
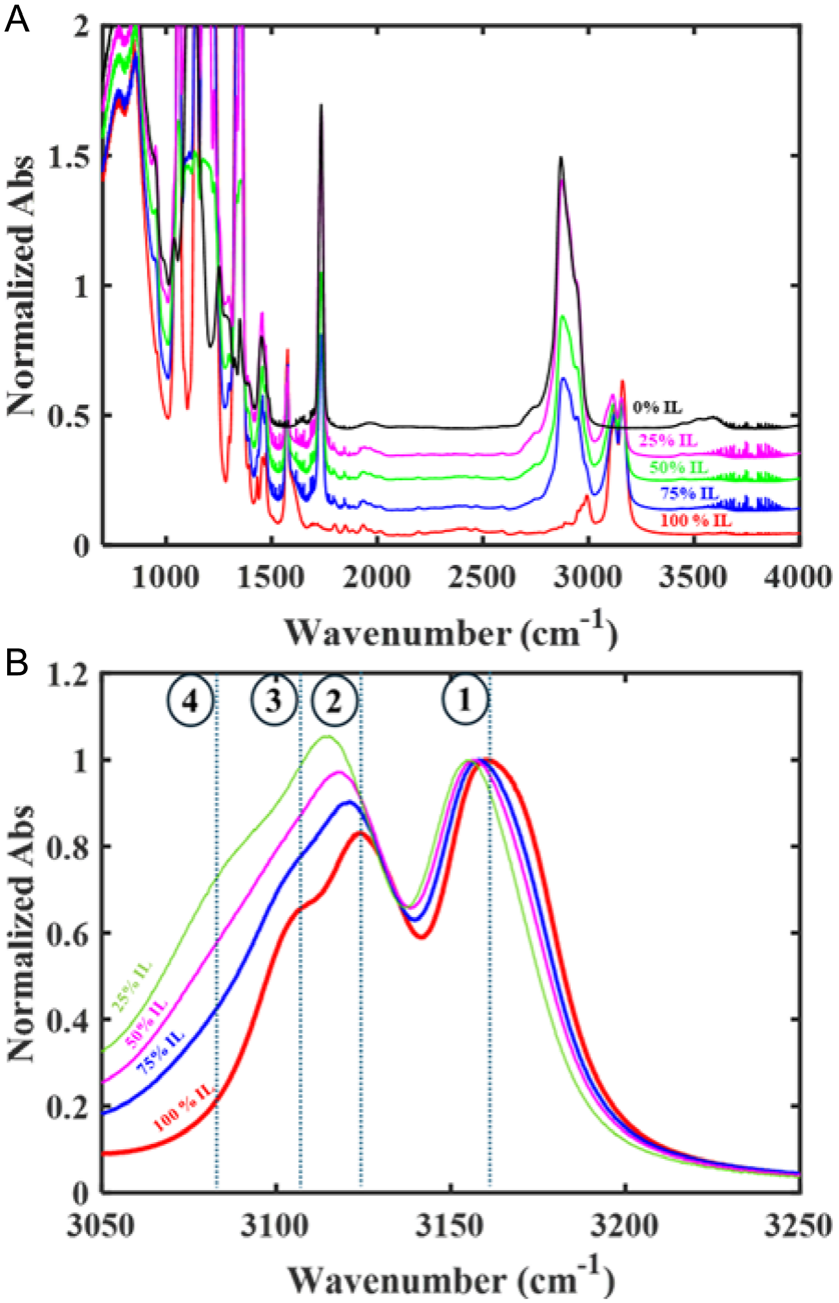
FTIR spectra of [C2C1Im][Tf2N]/cl‐PEGDA iongels at different IL loadings in (A) the mid‐IR range (700 to 4000 ${\mathrm{cm}}^{-1}$) and (B) the aromatic CH‐stretching range (3050 to 3250 ${\mathrm{cm}}^{-1}$) normalized to band 1. Bands 2 and 3 shift to lower wavenumbers as the fraction of IL decreases. A new feature, band 4, increases as the PEGDA content of the iongel increases.

As PEGDA is added to [C2C1Im][Tf2N], new vibrational bands appear in the CH‐stretching region (Figure 1). With increasing PEGDA concentration, the carbonyl stretch at 1730 ${\mathrm{cm}}^{-1}$ grows, while the aromatic CH‐stretching peaks, 3100 to 3200 ${\mathrm{cm}}^{-1}$, and the ring modes, 1500 to 1600 ${\mathrm{cm}}^{-1}$ of the imidazolium cation shrink (Figure 1A). In the pure IL, the aromatic CH‐stretching region of the spectra contains three major features (Figure 1B, bands 1, 2, and 3) [59]. As the PEGDA content increases, bands 1 and 2 shift, and a new feature appears at 3085 ${\mathrm{cm}}^{-1}$ (Figure 1B, peak 4). Feature 2 grows with increasing PEGDA concentration in the normalized spectra (Figure 1B). In contrast, the vibrational bands of the anion [47] do not shift (Figure S1). All these findings suggest an interaction between the imidazolium cation and PEGDA that changes with PEGDA content.

The acrylate groups of PEGDA do not cause the observed changes in the aromatic CH‐stretching bands of the cation. Polyethylene glycol (PEG), which lacks the acrylate group of PEGDA, causes similar changes in the CH‐stretching region as PEGDA (Figure S2). The peak frequency of the carbonyl stretching band (∼1730 ${\mathrm{cm}}^{-1}$, Figure S3) of the acrylate groups of the PEGDA remains unchanged with IL concentration. No interaction between the cation and the acrylate groups is detected.

### 2.2 FTIR of H/D Substituted Iongels

**FIGURE 2:**
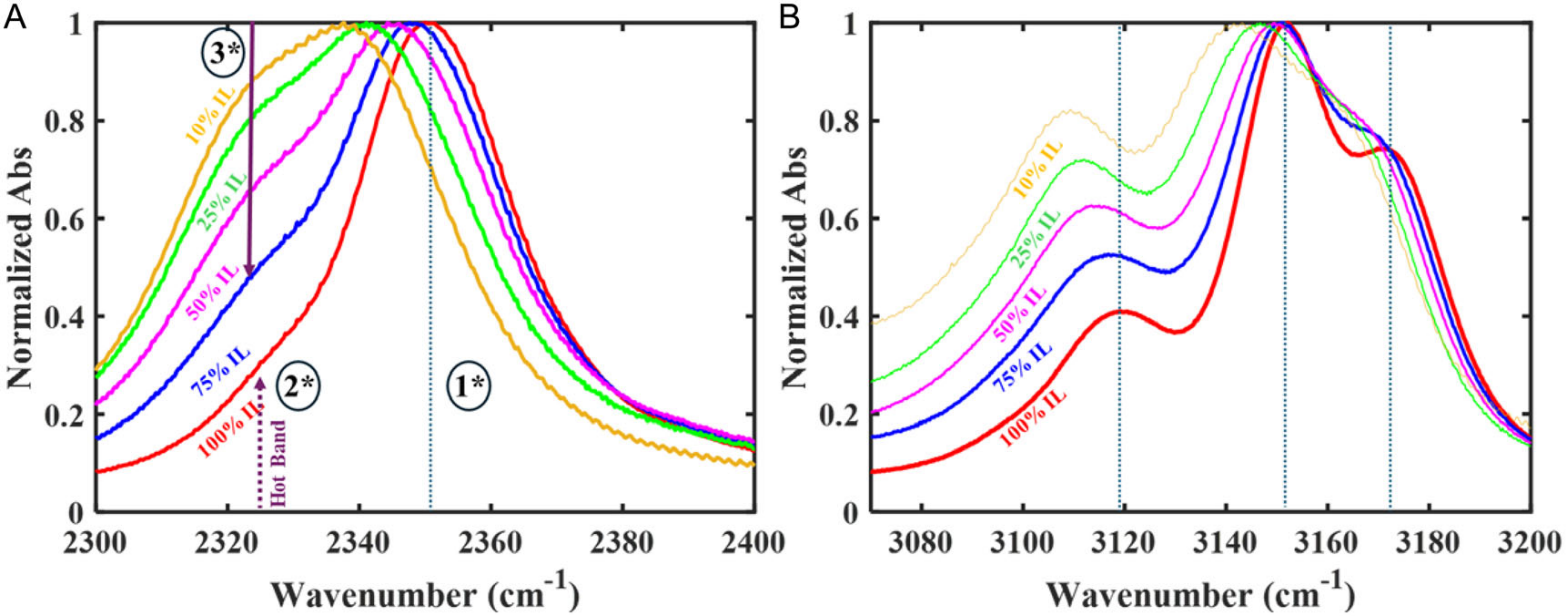
Normalized FTIR spectra of $C_{\left(2\right)}D$‐labeled [C2C1Im][Tf2N]/cl‐PEGDA at different IL loadings in the C‐D stretching region (A) and the C–H stretching region (B). A) In pure $C_{\left(2\right)}D$‐labeled [C2C1Im][Tf2N], the main band, 1*, is accompanied by a hot band, 2*. As PEGDA is added, a new feature, 3*, appears. B) The $C_{\left(4,5\right)}H$ stretching bands shift to lower wavenumbers as PEGDA content increases.

**FIGURE 3:**
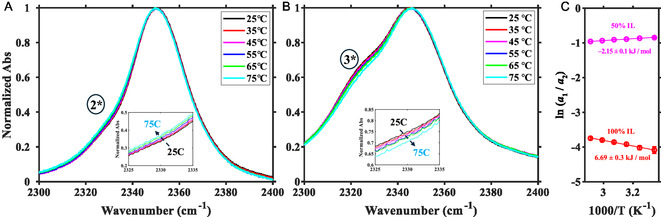
Temperature dependence of the $C_{\left(2\right)}D$ stretching band for of (A) pure $C_{\left(2\right)}D$‐labeled [C2C1Im][Tf2N] and (B) 50% v/v $C_{\left(2\right)}D$‐labeled [C2C1Im][Tf2N]/cl‐PEGDA from 25

Isotopic substitution moves the $C_{\left(2\right)}D$ stretch to a spectrally clear window (Figure 2A). The $C_{\left(2\right)}D$ stretch absorbs at 2350 ${\mathrm{cm}}^{-1}$ [61, 63], and $C_{\left(4,5\right)}D$ absorbs at 2390 ${\mathrm{cm}}^{-1}$, causing a small broad band in the baseline. The $C_{\left(2\right)}D$ spectrum also contains a small shoulder at 2325 ${\mathrm{cm}}^{-1}$. Several possible mechanisms could cause such a shoulder: a different solvation environment than the primary one, an overtone or a combination band, and the absorption by molecules that have a quantum of vibrational energy in a thermally excited low‐frequency mode that is anharmonically coupled to the IR active mode (i.e., a ‘hot‐band’). The amplitude of the 2* peak increases with increasing temperature while the 1* peak decreases (Figure 3A), which is the defining characteristic feature of a hot‐band. A van’t Hoff plot (Figure 3C, red) of the logarithm of the relative peak areas against inverse temperature gives an enthalpy change of 560±20 ${\mathrm{cm}}^{-1}$ (6.7±0.2 kJ ${\mathrm{mol}}^{-1}$). This value is close to the frequency of a low‐lying bending mode involving the $C_{\left(2\right)}$‐D and the ring. Anharmonic vibrational frequency analysis of deuterium substituted imidazolium cation predicts a vibration at 539 ${\mathrm{cm}}^{-1}$. Based on the results from DFT calculations and temperature‐dependent spectra, this low frequency (2325 ${\mathrm{cm}}^{-1}$) shoulder is assigned to the anharmonic coupling of a thermally excited bending mode to the $C_{\left(2\right)}D$ stretch.

C to 75

C. (C) The van’t Hoff plots for pure $C_{\left(2\right)}D$‐labeled [C2C1Im][Tf2N] (red) and 50% v/v $C_{\left(2\right)}D$‐labeled [C2C1Im][Tf2N]/cl‐PEGDA. The natural logarithm of the ratio of the area of bands 2* and 1* (red) and 3* and 1* (magenta) are plotted against inverse temperature, and the enthalpy change is determined from the slope of the line.

Varying the concentration of PEGDA in the iongels (Figure 2) causes two important changes in the CD‐stretching region (Figure 2A). The primary $C_{\left(2\right)}D$ stretching band, 1* [61, 63], shifts to lower frequency and a new peak, 3*, grows at 2,325 ${\mathrm{cm}}^{-1}$ (Figure 2A). The new band, 3*, appears on top of the hot‐band, 2*. Band 3* grows with increasing PEGDA content and suggests that the imidazolium has a different solvation environment than in pure $C_{\left(2\right)}D$‐labeled [C2C1Im][Tf2N]. As PEGDA is added to the neat IL, the ratio of the integral of band 3* to the integral of band 1* increases until 25% v/v [C2C1Im][Tf2N] and, then, plateaus at 1:2 (Figure S4). Bands 1* and 3* shift differently with composition. Band 3* is nearly constant with iongel composition; it shifts by < 2 ${\mathrm{cm}}^{-1}$ to lower wavenumbers, which suggests that it is due to a specific, concentration–independent interaction with PEGDA. Band 1*, however, shifts continuously > 10 ${\mathrm{cm}}^{-1}$ to lower wavenumbers as the PEGDA content increases. The significant redshift of band 1* is too large to be due to the dielectric response of the surroundings [72] because the dielectric constants of [C2C1Im][Tf2N] and PEGDA are so similar, ∼12 [73, 74]. Band 1* likely shifts due to local interactions around the cation, which are perturbed by the PEGDA, for example, restructuring of the ${\left[{\mathrm{Tf}}_{2}N\right]}^{-}$ or the nonspecific inclusion of PEGDA in the solvation shell of the imidazolium.

The temperature dependence of the 50% v/v $C_{\left(2\right)}D$‐labeled [C2C1Im][Tf2N]/cl‐PEGDA indicates that the 3* state is lower enthalpy than the 1* state. Band 3* decreases with increasing temperature, relative to band 1* (Figure 3B). A van’t Hoff analysis (Figure 3C, magenta) indicates an enthalpy change $\Delta H=-2.2\pm 0.1\;\mathrm{kJ}{\mathrm{mol}}^{-1}$, which is similar to a weak hydrogen bond [75, 76]. The entropy difference is $\Delta S=-14.1\pm 0.3\;J{\mathrm{mol}}^{-1}K^{-1}$. Representative plots (Figure S5) depict the goodness‐of‐fit.

The other two ring hydrogens, at the 4‐ and 5‐positions, also interact with the PEGDA chains, though less than the $C_{\left(2\right)}H$. In $C_{\left(2\right)}D$‐labeled [C2C1Im][Tf2N], the absorption features in the aromatic stretching range are due primarily to the $C_{\left(4\right)}H$ and $C_{\left(5\right)}H$ symmetric and antisymmetric stretches (3,150 and 3,175 ${\mathrm{cm}}^{-1}$, Figure 2B) and the remaining Fermi resonances (3,118 ${\mathrm{cm}}^{-1}$) [59, 60]. All three of these bands redshift 10 ${\mathrm{cm}}^{-1}$ as PEGDA content increases. The $C_{\left(2\right)}D$ stretching band redshifts 12 ${\mathrm{cm}}^{-1}$, which would be the equivalent of 17 ${\mathrm{cm}}^{-1}$ for a CH stretch, accounting for the lighter reduced mass. When the $C_{\left(2\right)}H$ is eliminated by methylation, that is, in 1‐ethyl‐2,3‐dimethylimidazolium ([C2C1C1Im] $\left[{\mathrm{Tf}}_{2}N\right]$), the remaining CH‐stretching bands shift similarly to $C_{\left(2\right)}D$‐labeled [C2C1Im][Tf2N] as the proportion of PEGDA increases (Figure S6). No equivalent to band 4 appears in the CH stretching regions of either $C_{\left(2\right)}D$‐labeled [C2C1Im][Tf2N]/cl‐PEGDA or [C2C1C1Im][Tf2N], also indicating that bands 4 and 3* are specific to the 2‐position.

### 2.3 NMR Spectroscopy

**FIGURE 4:**
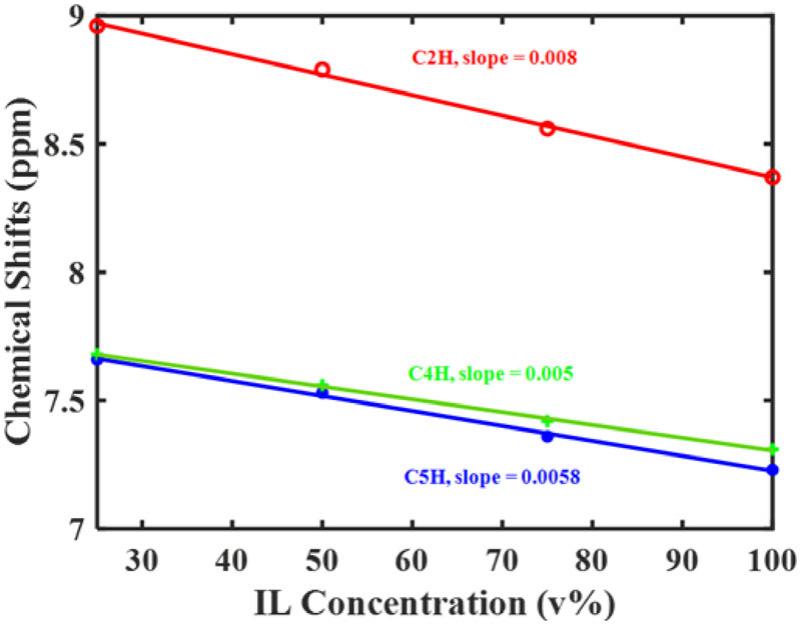
NMR chemical shifts for the three ring hydrogens of imidazolium as a function of the composition of the [C2C1Im][Tf2N]/PEGDA mixtures, $C_{\left(2\right)}H$ (red), $C_{\left(4\right)}H$ (green), and $C_{\left(5\right)}H$ (blue). $C_{\left(2\right)}H$ is the most sensitive to the composition of the mixture.

All three ring hydrogens (2‐, 4‐, and 5‐positions) of the imidazolium cation move downfield in the 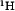 NMR spectra as the PEGDA concentration increases (Figure 4 and Figure S7). The chemical shift of the three ring hydrogens changes with varying PEGDA concentration and changes the most for $C_{\left(2\right)}H$. This trend agrees with the vibrational frequency shifts — $C_{\left(2\right)}D$ bands shift more than the $C_{\left(4,5\right)}H$ bands. Linear fits of the chemical shifts of these protons versus volume percent give slopes of $8.0\times 10^{-3}$ ($C_{\left(2\right)}H$, red), $5.0\times 10^{-3}$ ($C_{\left(4\right)}H$, green), and $5.8\times 10^{-3}$ ($C_{\left(5\right)}H$, blue) with an uncertainties of less than 4%. Additionally, like FTIR, NMR spectroscopy indicates that all the three ring hydrogens interact with PEGDA molecules, which was also observed in a similar system, [C2C1Im][Tf2N]/poly(ethyl glycidyl ether) [77]. Rapid exchange (compared to the inherent NMR timescale) between two states can cause them to appear as a single band in an NMR spectrum. We interpret the change in chemical shift as the change in the population of these two solvation states in rapid exchange as the PEGDA content increases.

### 2.4 2D‐IR Spectroscopy

**FIGURE 5:**
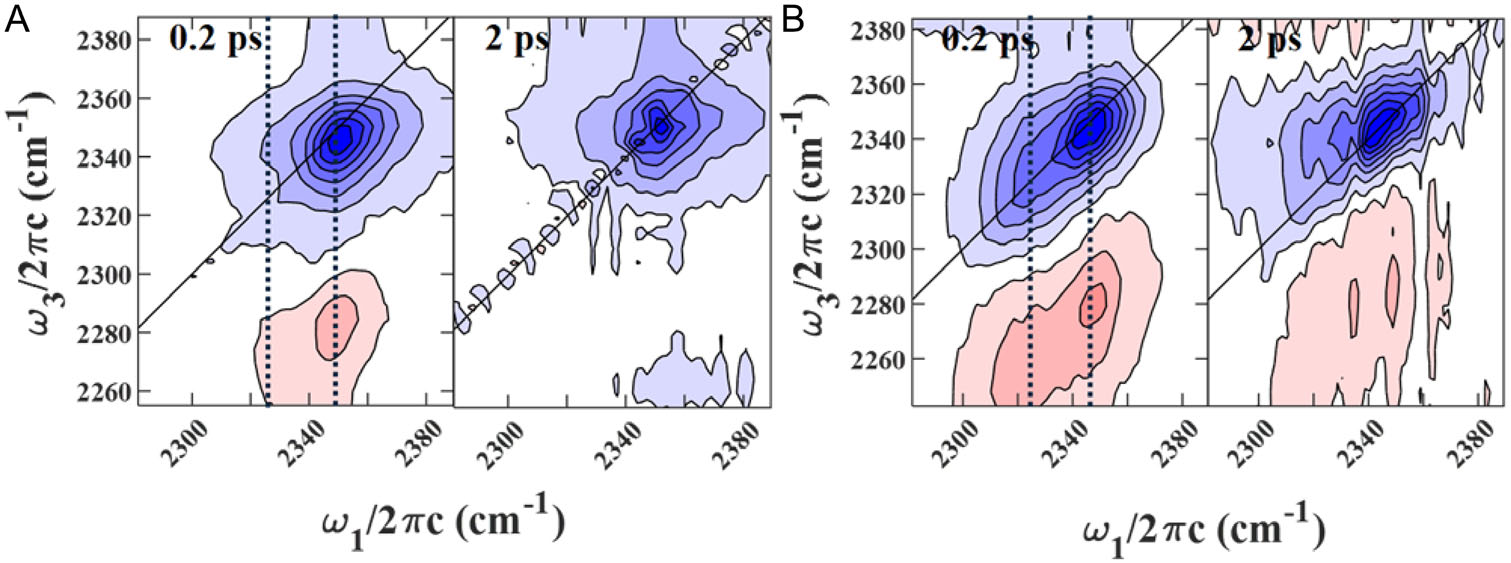
2D‐IR spectra of (A) pure $C_{\left(2\right)}D$‐labeled [C2C1Im][Tf2N] and (B) 50% v/v $C_{\left(2\right)}D$‐labeled [C2C1Im][Tf2N]/cl‐PEGDA at $t_{2}=0.2$ and 2 ps). The broken lines at $\omega_{1}=2325$ and 2350 ${\mathrm{cm}}^{-1}$ guide the eye. The pump‐probe signal intensity for (A) and (B) were 1.0 and 0.55 mOD, respectively.

The CD‐stretching mode in $C_{\left(2\right)}D$‐labeled [C2C1Im][Tf2N] is clearly detected. The spectra at $t_{2}=0.2$ and 2 ps are comprised of ground state bleach/stimulated emission (GSB/SE) at $\left(\omega_{1},\omega_{3})=(2350\;{\mathrm{cm}}^{-1},2350\;{\mathrm{cm}}^{-1}\right)$, and excited state absorption (ESA) at (2,350, 2,285 ${\mathrm{cm}}^{-1}$); the anharmonicity is 65 ${\mathrm{cm}}^{-1}$ (Figure 5A).

We interpret some subtle features of the 2D‐IR spectra of $C_{\left(2\right)}D$‐labeled [C2C1Im][Tf2N] (Figure 5) based on the hot‐band assignment established through FTIR spectroscopy (Figure 3). At $t_{2}=0.2\;\mathrm{ps}$, a less prominent diagonal feature is observed at 2327 ${\mathrm{cm}}^{-1}$ and some intensity appears as a weak cross‐peak (2330, 2350 ${\mathrm{cm}}^{-1}$). By 2 ps, no diagonal feature is observed at 2327 ${\mathrm{cm}}^{-1}$ above the level of the signal due to scattered light. We hypothesize that these observations are the result of rapid vibrational relaxation from a low‐frequency, thermally excited state to the total vibrational ground state [35, 78]. An alternative explanation is that the hot band, which is not large, is obscured by residual pump‐scatter. Although this assignment is somewhat tentative, this interpretation fits the observations of the structure of the 2D‐IR spectra and the assignment of a hot‐band from FTIR and DFT calculations.

Vibrational relaxation of the CD‐stretch occurs rapidly, consistent with previous measurements [63]. The ESA decays on a 0.73±0.09 ps timescale (Figure S8). The GSB/SE decays similarly, 0.58±0.04 ps, but to an offset, which indicates that vibrational relaxation of the $C_{\left(2\right)}D$ stretch excites inter‐ or intramolecular low‐frequency modes that are anharmonically coupled to it. This ‘hot ground state’ likely recovers on the timescale of thermal equilibration, which is much slower than our experimental time window.

The addition of PEGDA to $C_{\left(2\right)}D$‐labeled [C2C1Im][Tf2N] causes a second diagonal feature at 2325 ${\mathrm{cm}}^{-1}$ to appear, in addition to a 3 ${\mathrm{cm}}^{-1}$ redshift of the 2350 ${\mathrm{cm}}^{-1}$ diagonal feature (Figure 5B, broken lines), which is consistent with the FTIR spectrum. The 2325 ${\mathrm{cm}}^{-1}$ band's anharmonicity, 70 ± 2 ${\mathrm{cm}}^{-1}$, is 4 ${\mathrm{cm}}^{-1}$ larger than the main band, 66 ± 2 ${\mathrm{cm}}^{-1}$. Both the redshift and the increase in anharmonicity indicate a more anharmonic C‐D potential surface and stronger intermolecular interactions.

**FIGURE 6:**
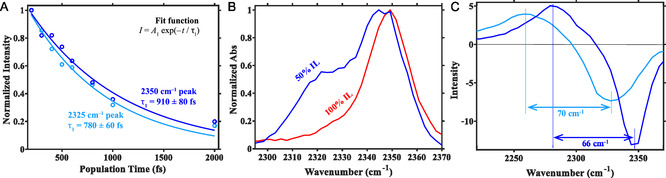
Selected features of the 2D‐IR spectra of pure $C_{\left(2\right)}D$‐labeled [C2C1Im][Tf2N] and 50% v/v $C_{\left(2\right)}D$‐labeled [C2C1Im][Tf2N]/cl‐PEGDA. (A) The decay of the ESA of the two bands (2325 and 2350 ${\mathrm{cm}}^{-1}$) in 50% v/v $C_{\left(2\right)}D$‐labeled [C2C1Im][Tf2N]/cl‐PEGDA. The vibrational relaxation times (910±80 fs for 2350 ${\mathrm{cm}}^{-1}$ peak and 780±60 fs for 2325 ${\mathrm{cm}}^{-1}$ peak) were extracted from the ESA features as the decay of the diagonal features (ground state bleach and stimulated emission) are affected by the hot ground states. (B) Diagonal cuts of the 2D‐IR spectra of $C_{\left(2\right)}D$‐labeled [C2C1Im][Tf2N] (red) and 50% v/v $C_{\left(2\right)}D$‐labeled [C2C1Im][Tf2N]/cl‐PEGDA) (blue). (C) Pump cuts for 50% v/v $C_{\left(2\right)}D$‐labeled [C2C1Im][Tf2N]/cl‐PEGDA) at two pump frequencies (2325 and 2350 ${\mathrm{cm}}^{-1}$) and plotted against probe frequencies ($\omega_{3}$). The anharmonicity of the 2350 ${\mathrm{cm}}^{-1}$ band is 4 ${\mathrm{cm}}^{-1}$ larger than the 2350 ${\mathrm{cm}}^{-1}$ band.

The CD‐stretching mode relaxes on a sub‐picosecond timescale (Figure 6A), and the relaxation dynamics of the 2325 ${\mathrm{cm}}^{-1}$ feature is slightly faster, 0.78±0.06 ps, than the 2350 ${\mathrm{cm}}^{-1}$ feature, 0.91 ± 0.08 ps, for the 50% v/v $C_{\left(2\right)}D$‐labeled [C2C1Im][Tf2N]/cl‐PEGDA iongel. Similar fast relaxation dynamics for the CD‐stretching mode has been reported for [C2C1Im] tris(pentafluoroethyl) trifluorophosphate [61]. The faster relaxation dynamics of the lower frequency mode in 50% v/v $C_{\left(2\right)}D$‐labeled [C2C1Im][Tf2N]/cl‐PEGDA also indicates stronger interaction between the cation and PEGDA chains compared to the cation–anion interaction.

The concentration ratio of the species that cause bands 1* and 3* in 50% v/v $C_{\left(2\right)}D$‐labeled [C2C1Im][Tf2N]/cl‐PEGDA can be estimated from the combination of linear and 2D‐IR spectra [79]. The ratio of the bands in the linear and 2D‐IR spectra can uniquely determine the concentration ratio ($C_{\mathrm{ratio}}$) of two components, even when their molar extinction coefficients are unknown [79]. If the molar extinction coefficient of a substance is known, Beer's law analysis can determine its concentration straightforwardly. When a substance partitions into two chemically distinct states, however, a Beer's law analysis will fail if hydrogen‐bonding interactions and other non‐Condon effects change the transition dipole. These effects make both the concentration and the extinction coefficients unknown, rendering the problem underdetermined. In principle, because the linear and third‐order signals in FTIR and 2D‐IR spectroscopy scale differently with transition dipole ($\mu^{2}$ and $\mu^{4}$, respectively), the ratio of the linear signal squared to the nonlinear signal eliminates the unknown transition dipole moment and allows the concentration to be determined unambiguously [79]. In practice, the 2D‐IR signal is also modulated by a variety of other factors, such as the lineshape, population and orientational relaxation, and the cancellation of GSB/SE and peaks. Following Donaldson [79], the corrected concentration ratio is

(1) $$C_{\mathrm{ratio}}=\frac{C_{a}}{C_{b}}=\frac{A_{a}^{2}S_{b}F_{a}}{A_{b}^{2}S_{a}F_{b}},$$

where $C_{i}$ is concentration, $A_{i}$ is linear absorbance, and $S_{i}$ is the 2D‐IR signal intensity of species i. In Equation (1), $F_{i}$ corrects for all the phenomena that modulate the 2D‐IR intensity,

(2) $$\begin{array}{cc} F_{i} & =R_{i}(T_{1},\tau_{\mathrm{rot}})\;\chi_{i}(\Delta_{\mathrm{an}},\Delta \omega_{\mathrm{hom}})\;B_{i}(\Delta \omega_{\mathrm{hom}},\Delta \omega_{\mathrm{inh}})\\ & \;\times \eta_{i}(A_{\mathrm{total}}(\omega_{i}))\;I_{\mathrm{laser}}(\omega_{i})\;G_{\mathrm{inst}}. \end{array}$$

$R_{i}$ corrects the reduction in signal due to vibrational and rotational relaxation and is a function of the population relaxation time, $T_{1}$, and rotational relaxation time, $\tau_{\mathrm{rot}}$; $\chi_{i}$ corrects the reduction due to peak overlap and is a function of the anharmonicity, $\Delta_{\mathrm{an}}$, and homogeneous linewidth, $\Delta_{\mathrm{hom}}$; $B_{i}$ corrects for lineshape effects and is a function of homogeneous and inhomogeneous linewidths, $\Delta_{\mathrm{hom}}$ and $\Delta_{\mathrm{inh}}$, respectively; η corrects for pump absorption due to the total absorption at the frequency of band i, $A(\omega_{i})$; $I_{\mathrm{laser}}(\omega_{i})$ corrects for the laser spectrum at the frequency of band i; and $G_{\mathrm{inst}}$ corrects for all other instrument specific factors. These factors can be determined from a series of 2D‐IR spectra and routine supplementary measurements like the laser spectrum. Donaldson [79] demonstrated that the concentration ratio of two components in a mixture can be estimated accurately when each of them possesses a well‐separated band in the FTIR and 2D‐IR spectrum [79].

The 2D‐IR experiments used the magic angle polarization (54.7 between the pump and probe pulses), which eliminates the effect of rotational dynamics on the signal intensity, that is, $R(T_{1},\tau_{\mathrm{rot}})=R(T_{1})$. The $G_{\mathrm{inst}}$ terms cancel because the 2D‐IR experiments were performed on a single instrument and with identical conditions. Thus, the F‐factor in our calculation becomes a product of $R(T_{1})$, $\chi (\Delta_{\mathrm{an}}$, Δω), $B(\Delta \omega_{\mathrm{hom}}$, $\Delta \omega_{\mathrm{inh}}$), $\eta (A_{\mathrm{total}})$ and $I_{\mathrm{laser}}(\omega )$. Each parameter of the F‐factor was evaluated as prescribed [79]. Briefly, $R(T_{1})$ values were estimated by extrapolating the vibrational relaxation dynamics of each GSB/SE feature to the $t_{2}=0\mathrm{ps}$ for the 50% v/v $C_{\left(2\right)}D$‐labeled [C2C1Im][Tf2N]/cl‐PEGDA iongel. $\chi (\Delta_{\mathrm{an}},\Delta \omega_{\mathrm{hom}})$ was determined by fitting pump slices of the iongel and comparing the experimental amplitude with the amplitudes obtained from their fits. 2D‐Gaussian fit of the 2D‐IR data produced $B(\Delta \omega_{\mathrm{hom}},\Delta \omega_{\mathrm{inh}})$. $\eta (A_{\mathrm{total}})$ and $I_{\mathrm{laser}}(\omega )$ values for each peak were obtained from the linear FTIR spectrum and pump‐pulse spectrum of the 2D‐IR measurements, respectively. After applying this method, the concentration ratio, $C_{\mathrm{ratio}}$, between 2325 ${\mathrm{cm}}^{-1}$ feature and 2350 ${\mathrm{cm}}^{-1}$ feature) for the 50% v/v $C_{\left(2\right)}D$‐labeled [C2C1Im][Tf2N]/cl‐PEGDA iongel system is 0.40±0.2. Incorporation of the obtained concentration ratio ($C_{\mathrm{ratio}}$) in the linear concentration absorbance relation (Beer–Lambert law) results in a transition dipole ratio of 1.0 ± 0.2. This ratio between the two transition dipoles indicates that the interaction (hydrogen bonding) between imidazolium cation and PEGDA is weak [72, 80], which agrees well with the van’t Hoff analysis (Figure 3C). The method, although derived for two well separated bands, works reasonably for bands overlapping with each other, though with larger uncertainties.

The significant overlap between the two bands (∼2350 and 2325 ${\mathrm{cm}}^{-1}$) and the small contribution from the $C_{\left(4,5\right)}D$ stretching modes (∼2390 ${\mathrm{cm}}^{-1}$) in the FTIR spectrum of the CD‐stretching region dominates the uncertainty in the estimated concentration ratio using this method. The uncertainty in the concentration ratio was determined based on different fits of the FTIR spectrum, which can describe the CD‐stretching region reasonably well, for example, two or three Voigt functions. For the 2D‐IR spectrum, the contributions of the $C_{\left(4,5\right)}D$ stretching modes were insignificant as the pump's intensity around 2390 ${\mathrm{cm}}^{-1}$ was significantly less than it is around 2350 and 2325 ${\mathrm{cm}}^{-1}$.

### 2.5 DFT Calculations

For the free imidazolium cation the $C_{\left(2\right)}H$ stretch appears at 3166 ${\mathrm{cm}}^{-1}$ and shifts to the red by ∼100 ${\mathrm{cm}}^{-1}$ with addition of ${\left[{\mathrm{Tf}}_{2}N\right]}^{-}$ (Figure S9), in agreement with literature values [81, 82]. The mode shifts by ∼50 ${\mathrm{cm}}^{-1}$ further in the complex with diethyl ether, which corroborates the interpretation of band 3* in the FTIR spectra as being the result of hydrogen bonding between the $C_{\left(2\right)}D$ and the ether oxygens. Anharmonic vibrational frequency calculations of the gas‐phase imidazolium, with no interaction partner, estimate the anharmonicity of the $C_{\left(2\right)}D$ stretching mode as 50 ${\mathrm{cm}}^{-1}$. Though anharmonic vibrational frequency calculations of the complexes proved prohibitively expensive, this value is roughly consistent with the experimental values in of bands 1* (66 ${\mathrm{cm}}^{-1}$) and 3* (70 ${\mathrm{cm}}^{-1}$) and supports the interpretation that both the redshift and anharmonicity of the CH‐stretching bands increase as intermolecular attractions increase.

### 2.6 Proposed Molecular Picture

**FIGURE 7:**
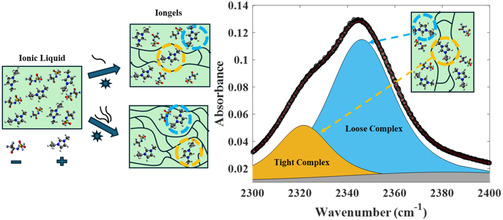
An illustration of the two different solvation environments for imidazolium cation, a tight complex with cl‐PEGDA (yellow) and loose complex with cl‐PEGDA (blue).

Imidazolium cations have two different solvation environments in [C2C1Im][Tf2N]/cl‐PEGDA iongels. In the first kind of environment, a loose complex (Figure 7, blue), the imidazolium has a bulk IL‐like environment and is weakly perturbed by the PEGDA. The $C_{\left(2\right)}D$ stretch has a characteristic frequency of 2350 ${\mathrm{cm}}^{-1}$. This environment changes gradually as the PEGDA content increases. In the second kind of environment, a tight complex (Figure 7, yellow), the imidazolium has a specific interaction with PEGDA. The $C_{\left(2\right)}D$ stretch has a characteristic frequency of 2325 ${\mathrm{cm}}^{-1}$. The environment is essentially unaffected by the average PEGDA composition. The tight complex has lower enthalpy than the loose complex, the $C_{\left(2\right)}D$ stretch frequency is redshifted compared to the loose complex, and the anharmonicity of the $C_{\left(2\right)}H$ stretch is larger. The redshift of the $C_{\left(2\right)}D$ stretch due to stronger interaction is consistent with earlier studies [63, 83]. These features all suggest that the tight complex comprises an imidazolium that hydrogen bonds at the 2‐position to the ethereal oxygens of PEGDA. Hydrogen‐bonded complexes of imidazolium and crown ethers have been crystallized [24]. The proton chemical shifts and DFT calculations are also consistent with shifting populations under rapid exchange.

The MD simulations by Son et al. [19] proposed two structural motifs for PEO in [C2C1Im][BF4]. In the model, PEO chains adopt both extended and collapsed conformations. The extended structures are nonspecifically solvated by the surrounding IL. On the other hand, the collapsed structures ‘wrapped’ the imidazolium cation. The wrapped conformation resembles the tight complex in [C2C1Im][Tf2N]/cl‐PEGDA, and the extended conformation resembles the loose complex. The wrapped conformation isolates the imidazolium from its surroundings, just as the tight complex is insensitive to PEGDA volume fraction. Conversely, the extended conformation leaves nearby imidazolium cations exposed to the surroundings, just as the loose complex senses the composition variation.

Additionally, the equilibrium between the extended and collapsed PEO chains in cl‐PEGDA at different IL‐PEGDA compositions may control the equilibrium between the tight and loose complexes in [C2C1Im][Tf2N]/cl‐PEGDA. The fraction of tight complexes grows with PEGDA concentration but saturates at a 1:2 ratio of tight to loose complexes at 20% v/v IL. A simple 1:1 binding model would predict that the fraction of tight complexes would continue to increase as the PEDGA content increases. We speculate that the solvation of the PEO chains changes from mostly IL to mostly polymer at low IL composition. Pure PEO is semicrystalline, and the chains tend to extend and pack efficiently. Perhaps this reduces the effective number of chains in collapsed conformations, which presumably are required to form tight complexes. If this proposal is correct, PEGDA with longer PEO chains, which have a greater conformational entropy, will also have, in their loops and turns, a greater fraction of tight complexes, especially at low IL loadings.

Finally, the resulting physical picture suggests an origin to the composition dependent dynamics of ${\mathrm{CO}}_{2}$ observed in 2D‐IR spectroscopy in this iongel. Kelsheimer [84] noted that the dynamics of ${\mathrm{CO}}_{2}$ in 20% v/v [C2C1Im][Tf2N]/cl‐PEGDA was slower than both the neat cl‐PEGDA and the [C2C1Im][Tf2N]. We speculate that the observed tight complexes may cause slower structural reorganization around both the imidazolium cation and the PEGDA chains and, ultimately, around the ${\mathrm{CO}}_{2}$. Testing the effect of tight and loose complexes on the dynamics of ${\mathrm{CO}}_{2}$ in these iongels is underway.

## 3 Conclusion

FTIR and 2D‐IR, supported by DFT calculations, reveal that imidazolium cations interact with PEGDA chains in two different modes, tight and loose complexes, in the iongel [C2C1Im][Tf2N]/cl‐PEGDA. NMR spectroscopy, overall, strengthens the FTIR and 2D‐IR observations such as stronger interaction between the imidazolium cation and PEGDA chains than the interaction between the imidazolium cation and the anion, although it could not disentangle the two different complexes. The aromatic C–H stretching bands of the imidazolium ion systematically shift to lower wavenumbers as the PEGDA concentration increases, and a new band appears (3085 ${\mathrm{cm}}^{-1}$). Isotopic substitution at the 2‐position moves the $C_{\left(2\right)}H$‐stretch to a clear spectral region and simplifies its structure. The $C_{\left(2\right)}D$ stretch (2350 ${\mathrm{cm}}^{-1}$) is accompanied by a hot‐band (2325 ${\mathrm{cm}}^{-1}$) due to anharmonic coupling of the $C_{\left(2\right)}D$ stretch with a thermally excited low‐frequency mode. In the presence of PEGDA, the imidazolium forms tight and loose complexes with a characteristic $C_{\left(2\right)}D$ frequency (2325 and 2350 ${\mathrm{cm}}^{-1}$, respectively). The anharmonicity of the $C_{\left(2\right)}D$ stretches of the tight and loose complexes is 70 ± 2 ${\mathrm{cm}}^{-1}$ and 66 ± 2 ${\mathrm{cm}}^{-1}$, respectively. The tight complex has lower enthalpy than the loose complex ($\Delta H=-2.2\;\mathrm{kJ}{\mathrm{mol}}^{-1}$). Cross‐linking of the PEGDA does not impact the imidazolium–PEGDA interaction detectably. The tight complex is consistent with the cation donating a hydrogen bond to the ether oxygens of the PEGDA chains primarily at the 2‐position and less so 4‐ and 5‐positions.

## 4 Experimental

### 4.1 Materials and Sample Preparation

1‐Ethyl‐3‐methylimidazolium bis(trifluoromethylsulfonyl)imide ([C2C1Im][Tf2N], Iolitec, > 99.5%, PubChem CID 53 384 303), 1‐ethyl‐2,3‐dimethylimidazolium ([C2C1C1Im]) $\left[{\mathrm{Tf}}_{2}N\right]$ (Iolitec, > 99%, PubChem CID 21 932 259), and poly(ethylene glycol)diacrylate (PEGDA, Mn 700, Sigma–Aldrich, CAS 26 570‐48−9) were vacuum dried (IL at 75

C, PEGDA at 23

C) before further use. The photo‐initiator (2,2‐Dimethoxy‐2‐phenylacetophenone, Aldrich, 99%, PubChem CID 90 571), poly(ethylene glycol) (Sigma–Aldrich, Mn 600, PubChem SID 481 110 092), sodium hydroxide (NaOH, Spectrum, > 97%, PubChem CID 14 798), and deuterium oxide ($D_{2}O$, Cambridge Isotope Laboratory, > 99.9%, PubChem CID 24 602) were used as‐received.

The isotopic substitution at the 2‐position of the ring followed Refs. [85] and [86]. The vacuum‐dried [C2C1Im][Tf2N] was mixed with $D_{2}O$ in a 1:30 molar ratio within a reaction vessel under nitrogen atmosphere. A catalytic amount of NaOH was added to this mixture and then the mixture was stirred at 60

C for 24 h. At the end of the reaction, the solution separated into two phases, water‐rich (clear, top) and IL‐rich (white, bottom). The vessel was cooled in an ice bath for 15 min. The reaction vessel was rinsed with $D_{2}O$, and the two phases were separated using the separatory funnel. The IL‐rich phase was vacuum dried for 24 h. The extent and selectivity of isotope exchange were determined by NMR spectroscopy. The $C_{\left(2\right)}H$ peak (8.4 ppm) decreased to < 5% of its initial intensity, and the 4‐ and 5‐position hydrogens (∼ 7.25 ppm) retained 90% of their initial intensities (Supporting Information).

IL was added to PEGDA to achieve a concentration series of 0, 25, 50, 75, and 100% by volume (% v/v) [C2C1Im][Tf2N]. The mixtures were stirred for 12 h at room temperature to ensure complete mixing.

### 4.2 FTIR Spectroscopy

Linear IR spectra were recorded at 0.125 ${\mathrm{cm}}^{-1}$ resolution under nitrogen atmosphere using a Thermo Fisher Nicolet FTIR 6700 spectrometer. ∼5 μL of each sample was sandwiched between two CaF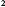 windows (Crystran, 25 mm × 2 mm UV grade) in a brass cell under nitrogen atmosphere. PTFE spacers (Harrick Scientific) set the path length to 12 μm for all the samples except the 10% v/v sample (25 μm). The samples cured for 3 min under UV‐light (36W UV‐lamp, Melody Susie). The water content of these samples was < 0.5 $\mathrm{mol}L^{-1}$, estimated from the OH‐stretching bands (3000 to 3700 ${\mathrm{cm}}^{-1}$) [87, 88]. Spectra were fit using multi‐Voigt or multi‐Gaussian fitting models in both Origin and MATLAB software.

### 4.3 2D‐IR Spectroscopy

The 2D‐IR spectrometer used in these experiments has been described in detail elsewhere [35, 89]. Briefly, output pulses (120 fs, 805 nm, and 15 nm bandwidth) of a commercial, 5 kHz Ti:sapphire laser (Coherent Legend Elite) were directed to a home‐built optical parametric amplifier [90] (BBO, CASTECH) and difference frequency generator (AgGaS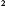, EKSMA optics) that produced tunable mid‐IR light (1.1μJ/pulse μJ/pulse, 200 ${\mathrm{cm}}^{-1}$ bandwidth). A fast‐scanning Mach‐Zehnder interferometer modulated the coherence time, $t_{1}$, between the pump pulses, while the population stage created the waiting time, $t_{2}$, between the pump and the probe pulses. For each $t_{2}$, the ‘initial’ frequency axis, $\omega_{1}$, was the Fourier conjugate of $t_{1}$, and the ‘final’ frequency axis, $\omega_{3}$, was measured directly in a spectrograph (Jobin‐Yvon iHR320, 75 lines/mm) by dispersing the third‐order signal and the probe pulse onto a 2 × 32‐element mercury cadmium telluride (MCT) detector (Infrared System Development, liquid $N_{2}$ cooled). The signal‐to‐noise (S/N) ratio ranges from 35 to 10 depending on the waiting time and the system we are investigating. The time to acquire each 2D‐IR spectrum at different waiting times (and for different systems) ranges over an hour to 3.5 h, depending on the number of scans. MATLAB (MathWorks) [91] was used to record, Fourier transform, phase correct, and analyze the collected third‐order signal. All 2D‐IR spectra were recorded at 23°C.

### 4.4 NMR Spectroscopy


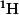
 NMR spectra were recorded on a Bruker Avance III HD 500 spectrometer and analyzed in TopSpin software (Bruker). 0.5 mL of each sample was taken in a 5 mm outer diameter NMR tube (Wilmad) fitted with a coaxial insert (Wilmad WGS‐5BL) containing $D_{2}O$, for a frequency lock and external reference [92].

The FTIR spectra of cross‐linked and non‐cross‐linked iongels (Figure S10), suggests that cross‐linking does not make any significant changes in [C2C1Im][Tf2N]–PEGDA interaction. None of the prominent IR bands, for example, the CH‐stretching modes (3050 to 3200 ${\mathrm{cm}}^{-1}$) or the ring modes (1500 to 1600 ${\mathrm{cm}}^{-1}$), of the cation shift due to cross‐linking, only the intensity drops slightly. Based on this finding, all the NMR measurements were carried out without UV‐curing the mixtures.

### 4.5 Electronic Structure Calculations

Gas phase density functional theory (DFT) calculations, in Gaussian16 [93], employed the B3LYP hybrid density functional and the 6‐31++G(d, p) basis set [81, 87, 94]. The geometries of the individual ions (molecules) were optimized, the fragments were combined to form the ion pair (ion‐molecule adduct), and then their geometries were re‐optimized. For simplicity, the PEGDA was modeled by diethyl ether. Both harmonic (for all the systems) and anharmonic (only for imidazolium cation, keyword: Freq=Anharm) frequencies were extracted using the same method. The harmonic IR frequencies were scaled by a factor 0.96 [48, 87].

## Supporting Information

Additional supporting information can be found online in the Supporting Information section.

## Author Contributions

**Kallol Mukherjee**: data curation (lead), investigation (equal), writing – original draft (equal), writing – review and editing (supporting). **Matthew R. Liberatore**: investigation (supporting), writing – review and editing (supporting). **Tyler A. Parrack**: investigation (supporting), writing – review and editing (supporting). **Sean Garrett‐Roe**: conceptualization (lead), investigation (equal), writing – original draft (equal), writing – review and editing (lead).

## Funding

This study was supported by Basic Energy Sciences (DE‐SC‐0023474).

## Conflict of Interest

The authors declare no conflicts of interest.

## Supporting information

Supplementary Material

## Data Availability

The data that support the findings of this study are available in the supplementary material of this article.
