# Supplementary material for: Structure and Dynamics of Imidazolium in an Ionic Liquid‐PEGDA Iongel via IR, 2D‐IR, and NMR Spectroscopy
Source: Chemphyschem. 2026 Apr 19;27(8):e202500925. doi: 10.1002/cphc.202500925 (PMC13092334; doi:10.1002/cphc.202500925)
Supplement: Supplementary file 1 — Supplementary Material [file CPHC-27-e202500925-s001.pdf]

Structure and Dynamics of Imidazolium in an Ionic Liquid-PEGDA Iongel via IR, 2D-IR, and NMR Spectroscopy

Kallol Mukherjee, Matthew R. Liberatore, Tyler A. Parrack, and Sean Garrett-Roe*

Department of Chemistry, University of Pittsburgh, Pittsburgh, PA, USA

E-mail: sgr@pitt.edu

S1 Characterization of $C_{(2)}$D-labeled $[C_2C_1Im][Tf_2N]$

Characterization of $C_{(2)}$D-labeled $[C_2C_1Im][Tf_2N]$ was carried out using NMR spectroscopy. The figure shown (Scheme S1) below indicates the conversion efficiency. The peak at 8.4 ppm (boxed by broken lines), which corresponds to $C_{(2)}$H of the cation, diminishes 98%. The other two ring hydrogens remain intact up to 90%.

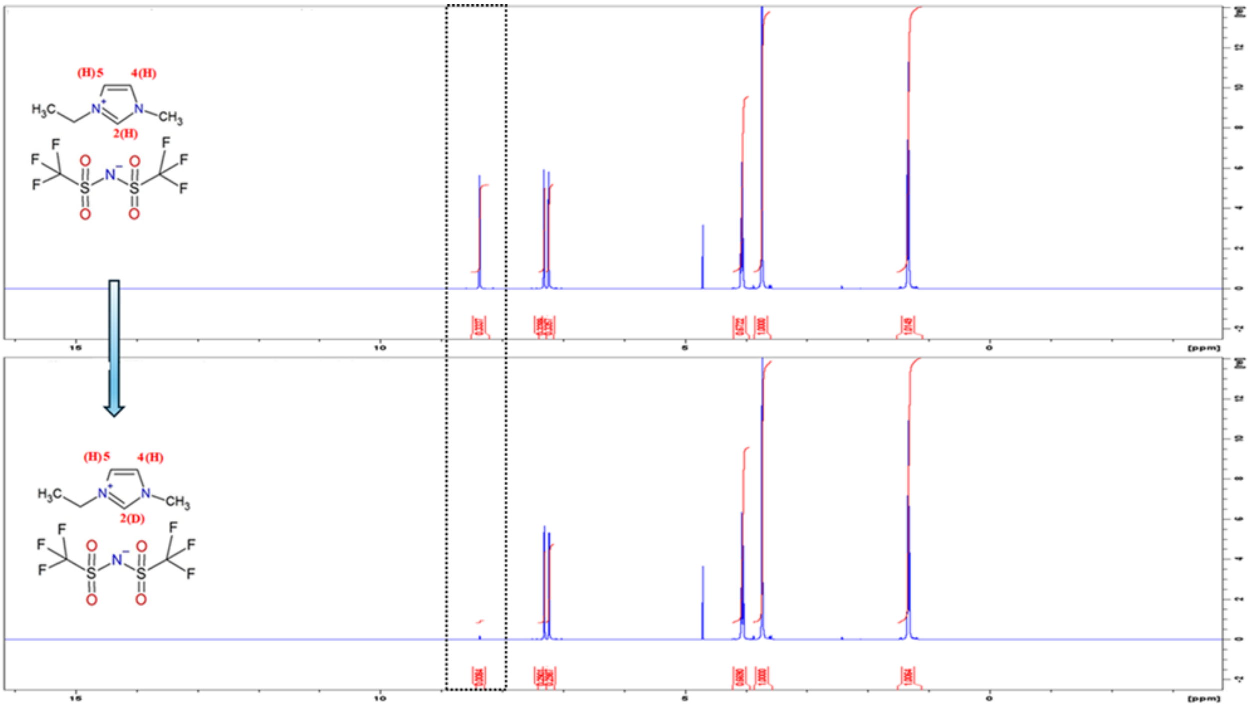

Scheme S1: Characterization of H/D exchange in $[C_2C_1Im][Tf_2N]$.

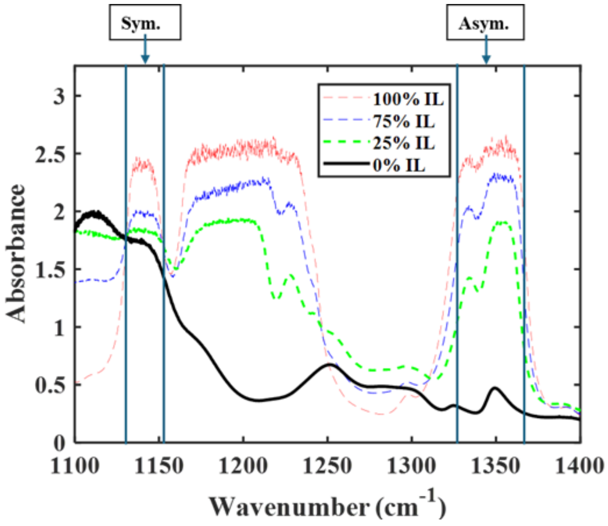

Figure S1: Sulfonyl stretching bands, symmetric ($1130\,cm^{-1}$ to $1155\,cm^{-1}$, boxed) and asymmetric ($1330\,cm^{-1}$ to $1370\,cm^{-1}$, boxed) of the anion at different IL loadings.[1] The peaks saturate but do not shift.

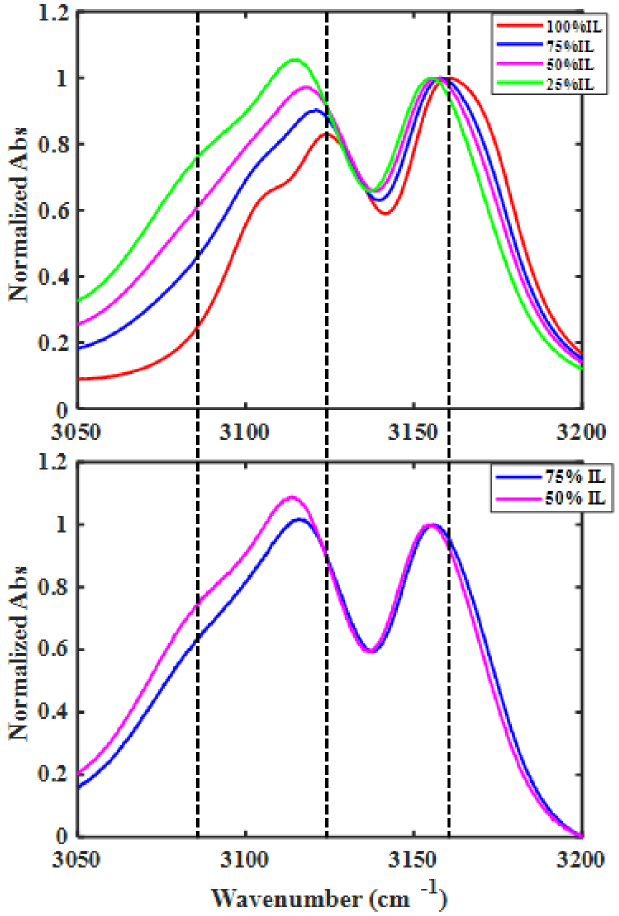

Figure S2: C-H stretching absorption bands (3050 cm^{-1} to 3200 cm^{-1}) of imidazolium cation at different $[C_2C_1Im][Tf_2N]$ loadings for $[C_2C_1Im][Tf_2N]$/cl-PEGDA iongels (upper panel) and $[C_2C_1Im][Tf_2N]$/PEG iongels (bottom panel). Broken black lines indicate peak positions.

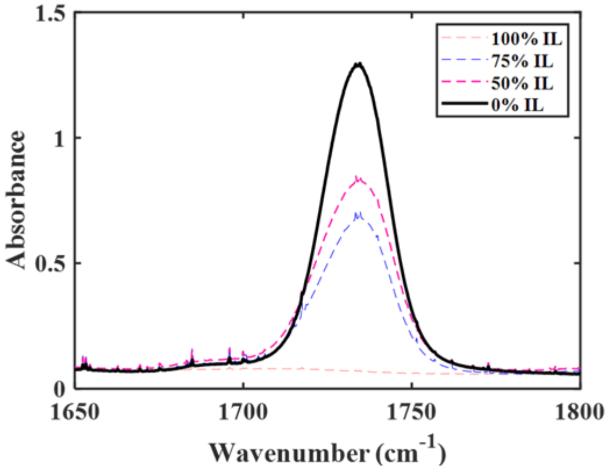

Figure S3: Carbonyl stretching bands, ranging from 1700 cm^{-1} to 1750 cm^{-1}, of PEGDA at different [C_2C_1Im][Tf_2N] loadings.[2] The peak frequency and shape do not change as a function of composition.

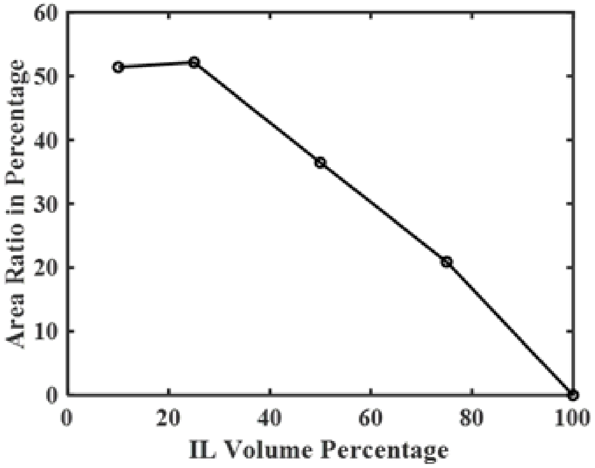

Figure S4: Ratio of the area under the peak at 2325 cm^{-1} to the area under the peak at 2350 cm^{-1} in percentage versus $C_{(2)}$D-labeled [C_2C_1Im][Tf_2N] volume percentage plot, indicating nonlinear growth of the new feature at 2325 cm^{-1}.

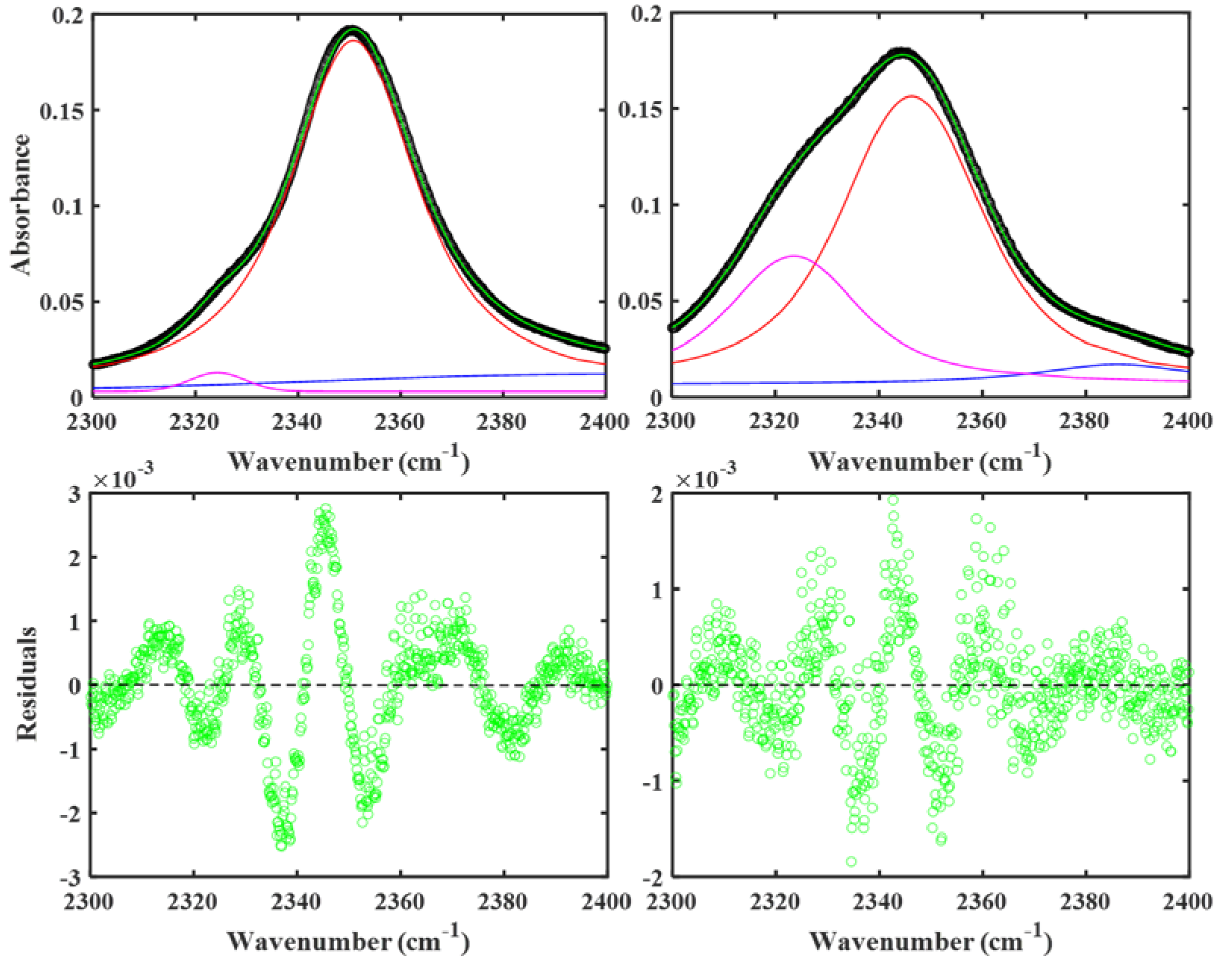

Figure S5: Representative fits (upper panel) and residuals (lower panel) for the FTIR spectra (at room temperature) of pure $[C_2C_1Im][Tf_2N]$ (left panel) and 50% v/v $[C_2C_1Im][Tf_2N]$/PEGDA mixture. In the upper panel solid lines (green) passing through the black open experimental data points indicates the cumulative fit while the other solid line plots of different colors (blue, magenta and red) indicates the contributions of the individual components.

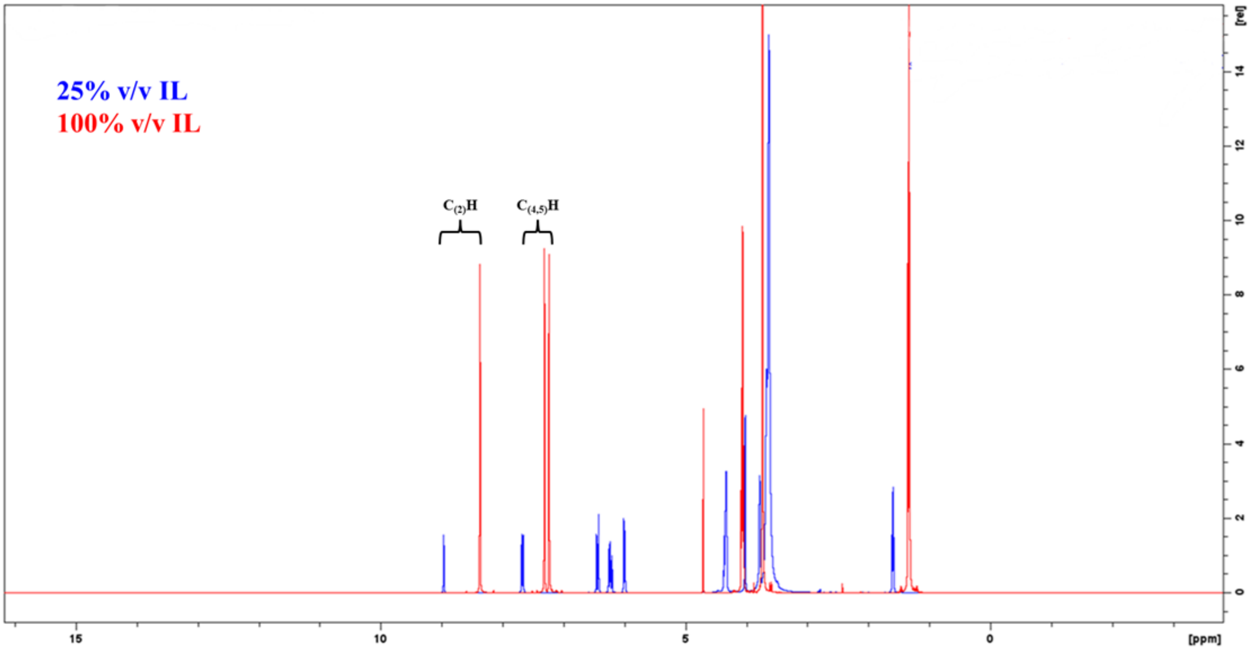

Figure S6: Proton NMR spectra of pure $[C_2C_1Im][Tf_2N]$ and 25% v/v $[C_2C_1Im][Tf_2N]$/PEGDA mixture. The one-sided curly braces indicate the $C_{(2)}H$, $C_{(4)}H$ protons

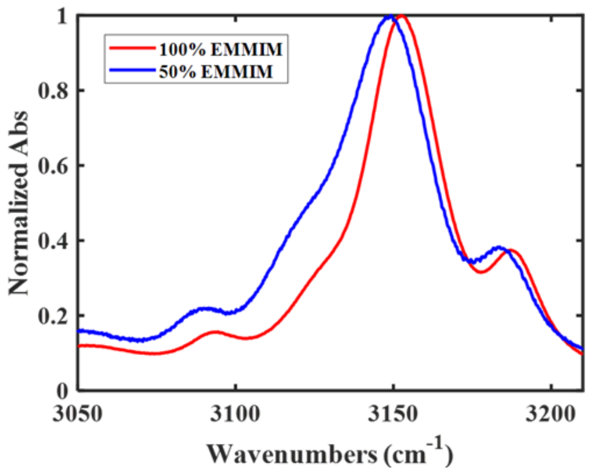

Figure S7: C-H stretching absorption bands (3050 cm^{-1} to 3200 cm^{-1}) of imidazolium cation at two different $[C_2C_1C_1Im][Tf_2N]$ loadings for $[C_2C_1C_1Im][Tf_2N]$/cl-PEGDA iongels.

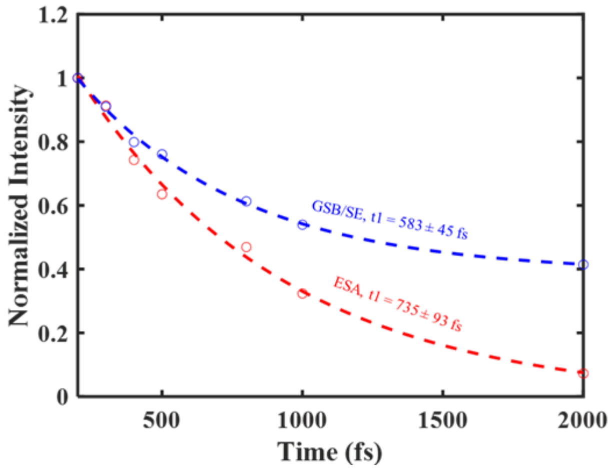

Figure S8: Comparison between vibrational relaxation of GSB/SE (blue) and ESA (red) features for pure $C_{(2)}D$-labeled $[C_2C_1Im][Tf_2N]$. Lines passing through the circular data points are the single exponential fits.

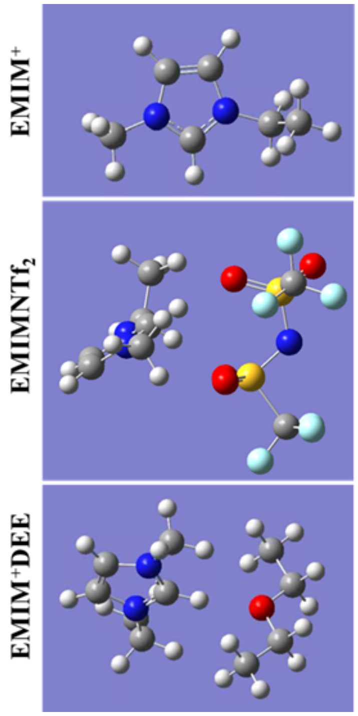

Mode	Frequency(cm^{-1})	Intensity
C2H str.	3166	31

Mode	Frequency(cm^{-1})	Intensity
C2H str.	3070	374

Mode	Frequency(cm^{-1})	Intensity
C2H str.	3023	311
	3025	68

Figure S9: Optimized structures of ion pairs and the tables beside each figure provide the calculated frequencies and intensities of the $C_{(2)}H$ stretching mode. The calculations are performed in gas phase.

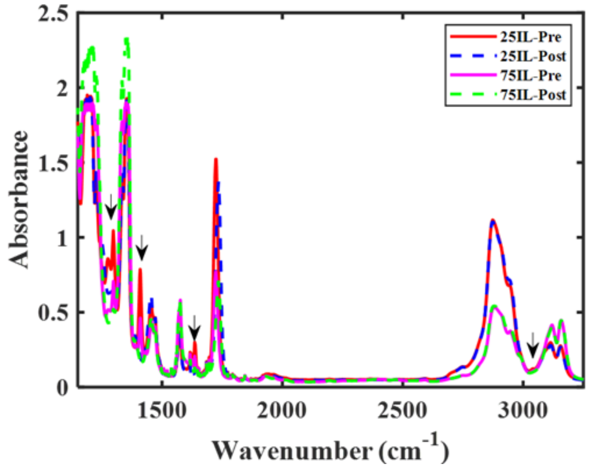

Figure S10: Comparison between $[C_2C_1Im][Tf_2N]$/cl-PEGDA iongels with two different $[C_2C_1Im][Tf_2N]$ loadings, 75% v/v and 25% v/v, before (pre) and after (post) UV-curing. Arrows indicate the peaks which confirm complete cross-linking.

References

(1) Noack, K.; Schulz, P. S.; Paape, N.; Kiefer, J.; Wasserscheid, P.; Leipertz, A. The role of the C2 position in interionic interactions of imidazolium based ionic liquids: a vibrational and NMR spectroscopic study. *Physical Chemistry Chemical Physics* **2010**, *12*, 14153, DOI: 10.1039/c0cp00486c.

(2) Martins, A. P. S.; Fdz De Añastro, A.; Olmedo-Martínez, J. L.; Nabais, A. R.; Neves, L. A.; Mecerreyes, D.; Tomé, L. C. Influence of Anion Structure on Thermal, Mechanical and CO2 Solubility Properties of UV-Cross-Linked Poly(ethylene glycol) Diacrylate Iongels. *Membranes* **2020**, *10*, 46, DOI: 10.3390/membranes10030046.
